# Routine Use of Color Doppler in Fetal Heart Scanning in a Low-Risk Population

**DOI:** 10.5402/2012/496935

**Published:** 2012-05-20

**Authors:** Torbjørn Moe Eggebø, Claudia Heien, Magne Berget, Christian Lycke Ellingsen

**Affiliations:** ^1^Department of Obstetrics and Gynecology, Stavanger University Hospital, N-4068 Stavanger, Norway; ^2^Department of Pediatrics, Stavanger University Hospital, N-4068 Stavanger, Norway; ^3^Department of Pathology, Stavanger University Hospital, N-4068 Stavanger, Norway

## Abstract

*Objectives*. To investigate the detection rate of major fetal heart defects in a low-risk population implementing routine use of color Doppler. *Material and Methods*. In a prospective observational study, all women undergoing fetal heart scanning (including 6781 routine examinations in the second trimester) during a three-year period were included. First a gray-scale scanning was performed including assessment of the four-chamber view and the great vessels. Thereafter three cross-sectional planes through the fetal thorax were assessed with color Doppler. *Results*. Thirty-nine fetuses had major heart defects, and 26 (67%) were prenatally detected. In 9/26 (35%) of cases the main ultrasound finding was related to the use of color Doppler. The survival rate of live born children was 91%. *Conclusions*. Routine use of color Doppler in fetal heart scanning in a low-risk population may be helpful in the detection of major heart defects; however, still severe malformations were missed prenatally.

## 1. Introduction

The incidence of cardiac defects is estimated to be 8 per 1000 births, and annually around 36,000 children are live born with heart defects in the European Union [1]. The incidence of major congenital cardiac defects is approximately 3-4 per 1000 live births and 5 per 1000 fetuses in the second trimester [2]. Prenatal diagnosis of major malformations by ultrasonography may lower the perinatal mortality [3, 4], allowing for better planning of the delivery and postnatal care [5]. The prenatal detection rate of major heart defects varies from 5% to 75% in low-risk populations [6–11]. The detection rate depends on the sonographers education and experience [12, 13]. Although it is possible to identify situations with increased risk for cardiac malformations, most fetal cardiac defects appear in the low-risk population, and the overall detection rate of major heart defects is still not satisfactory [14, 15]. The use of color Doppler is recommended when a heart defect is suspected [16], but the effectiveness of routine use in low-risk populations is not documented and remains controversial [17]. Routine use of color Doppler is not implemented in second trimester scanning guidelines from the International Society of Ultrasound in Obstetrics and Gynecology (ISUOG) [18]. In American Institute of Ultrasound in Medicine (AIUM) guidelines for 2011, color Doppler is regarded as an optional method, but recommended for suspected cardiac flow abnormalities [19]. For safety reasons routine use of pulsed color Doppler is advised against in the first trimester [20]. We aimed to investigate the detection rate of major heart defects in a low-risk population implementing the routine use of color Doppler in second and third trimester fetal heart scanning and to focus on the main ultrasound findings responsible for the detection of the anomalies.

## 2. Patients and Methods

### 2.1. Patients

 We performed a prospective observational study at the Stavanger University Hospital from May 2006 to July 2009. Routinely the use of color Doppler was implemented in fetal heart scanning in the second and third trimester. The ultrasound laboratory at the hospital is classified as a secondary-level unit. All pregnant women in Norway are recommended to have a second-trimester ultrasound scanning in pregnancy week 17–20, and the examination is free of charge. Around 60% of the women in the region attend the routine scan at the hospital, and the rest attend the scan at their private gynecologist. Private gynecologists in the region do not routinely use color Doppler. During the study period 6781 women attended a second trimester routine scan at the hospital. The local Ethics Committee approved the study, and all women attending the routine scan gave written informed consent.

### 2.2. Methods

 Six midwives educated for scanning performed the routine examinations using EUB Hitachi 5500 (Kashiwa, Japan), Voluson 730 Pro or Voluson 730 Expert (GE Medical Systems, Kretz, Austria) devices with 3.5–7.5-MHz multifrequency transabdominal transducers. Examinations before or after the routine scan were performed by physicians or midwives and only on medical indications. Before the start of the study, the midwives were briefly trained on using color Doppler. First an extended gray-scale fetal heart scanning was performed as recommended in ISUOG's guidelines [18]. Thereafter color Doppler was added, and three cross-sectional planes through the fetal thorax were assessed: the four-chamber, five-chamber, and the three-vessel trachea view as described by Yagel et al. [21]. A new scan was performed two weeks later if a proper visualization during the routine scan failed. If a fetal heart defect was suspected, an obstetrician performed a thorough fetal examination and fetal karyotyping was offered. In cases with uncertain diagnosis the women were referred to the National Center for Fetal Medicine in Trondheim for an extended examination.

### 2.3. Follow-Up

 In the geographical region there is only one labour ward with around 4500 deliveries yearly and one pediatric department. Heart surgery is not performed at the hospital, but the pediatric unit is responsible for the follow up of all patients. Children with intrauterine suspected heart malformations underwent echocardiography within the first days after delivery, and the final diagnoses were based on postnatal cardiac echocardiography and operative findings. If the pregnancy was terminated and in cases with intrauterine fetal death (IUFD), a thorough autopsy was performed and the findings were compared with the ultrasound findings. In three cases with termination of pregnancy (TOP), autopsy was not performed due to lack of consent from the parents. The ultrasound diagnoses from the National Centre for Fetal Medicine were then used as the final diagnosis. Women referred from private gynecologists because of suspected heart defects are not included in the study population.

### 2.4. Classification of Heart Defects

 The defects were classified as major when they were potentially lethal, when they were severe enough to warrant TOP, or when surgical repair was required and minor when no intervention was likely. The classification of major or minor heart defects in live born children was performed one year after the end of the inclusion period.

## 3. Results

Thirty-nine fetuses examined at the hospital during the study period had major heart defects, and 26 (67%) were prenatally detected. Of these, 19/39 (49%) were detected at the second trimester routine scan, 3/39 (8%), were detected before the routine scan and 4/39 (10%) were detected later in pregnancy. The karyotyping was abnormal in 10 cases. Of the prenatally detected fetuses, one died during pregnancy, one died short time after delivery, and in thirteen cases the pregnancy was terminated. Eleven children with prenatally diagnosed heart defects underwent open-heart surgery, and of those 64% (7/11), were referred to a center with heart surgery before labour. Details of the ultrasound findings, the final diagnosis, associated anomalies, and outcomes in prenatal diagnosed cases are presented in Table 1. One case with right aortic arch was also diagnosed, but not classified as a major heart defect.

There were no major discrepancies between ultrasound findings and autopsy or postnatal findings (Table 1). A major heart defect was confirmed in all cases admitted to the National Center for Fetal Medicine. In some cases the ultrasound examination at the tertiary center, the postnatal findings, or the autopsy gave additional information, leading to a more precise final diagnosis. There were no “false-positive” ultrasound findings leading to an unwarranted termination of pregnancy.

Thirteen cases included in the routine scan at the hospital turned out having prenatally undiagnosed major heart anomalies. Two died during pregnancy, one shortly after delivery, seven underwent open-heart surgery, and in three cases with moderate pulmonary stenosis balloon dilatation was performed. Detailed information is presented in Table 2.

In all, 23/39 (59%) children with major heart defects were born alive and the survival rate of live born children was 91%. Two children died shortly after birth, both with trisomy 13. The surgical repair was successful in all operated children, and they were in good condition one year after the operation.

The main ultrasound finding responsible for the spotting of the heart defect was unequal size of the ventricles in four cases, unilateral perfusion of right ventricle in three, common atrioventricular valve in three, atrioventricular insufficiency in four, overriding vessel in two, parallel vessels in three, one great vessel in “three-vessel view” in four, retrograde blood flow in “three-vessel view” in two, and abnormal position of the heart in one case (Table 1).

## 4. Discussion

The overall detection rate of major fetal heart defects including routine use of color Doppler was 67%, and 49% were detected at the routine second trimester scan. Our detection rate was higher than the results in previous Scandinavian studies, but still important malformations were missed. A Swedish study compared first and second trimester detection of fetal malformations, and 11% and 15% of major heart malformations were detected, respectively [10]. In a low-risk population from northern Norway, 24% of major heart defects were diagnosed prenatally [8], and in an unselected population examined at the Norwegian National Center for Fetal Medicine, 37% of major hear defects were detected at the routine scan, and the overall detection rate was 57% [9]. A study from a teaching hospital in London has reported a very high detection rate without routine use of color Doppler (75%) [6].

In the 1990s a systematic examination of the four-chamber view was the basic of the fetal heart scanning [22]. Examining the four-chamber view often fails to detect transposition of the great arteries (TGAs), tetralogy of Fallot, double-outlet right ventricle (DORV), truncus arteriosus communis, and interruption of the aortic arch (IAA) [7]. Assessment of the great vessels by using the three-vessel and trachea view as described by Yagel et al. [21] improves detection rates [6, 23–25]. ISUOG has published guidelines for the “basic” and the “extended basic” cardiac scan and for a fetal echocardiogram [16, 18]. Recently the three-vessel and trachea view with color flow mapping has been recommended [17, 23]. Using color Doppler is mandatory in examination of fetuses with suspected heart defects [16, 19]; however, the value of color Doppler in a low-risk populations is still not documented.

The higher detection rate in our study compared to other Scandinavian studies might be related to the use of color Doppler. However, the study has several limitations. It is performed on a low-risk population, but a selection bias is possible. Some women preferred the routine scan at the hospital, others at their private gynecologists. The examination is free of charge for all; thus, we think this selection is random. The quality of ultrasound devices improves continuously, and the results might be related to better equipment. Both gray-scale scanning and color Doppler were used in all examinations; thus, we cannot reliably distinguish between heart defects detected in gray-scale or by using color Doppler. Our study design was observational without any control group. The study was not designed to compare results from examinations inside and outside the hospital, but the detection rate was much lower in fetuses examined outside the hospital (3/16). A randomized controlled study is necessary before routine use of color Doppler eventually might be recommended.

**Figure 1:**
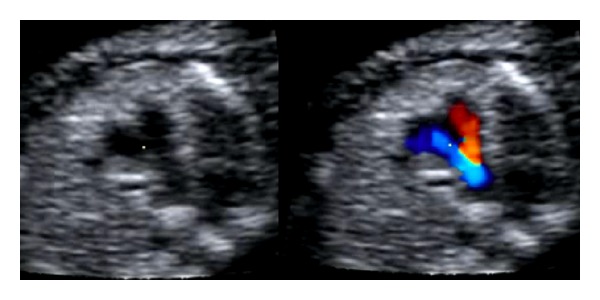
Three-vessel view illustrating retrograde blood flow in the pulmonary artery.

**Figure 2:**
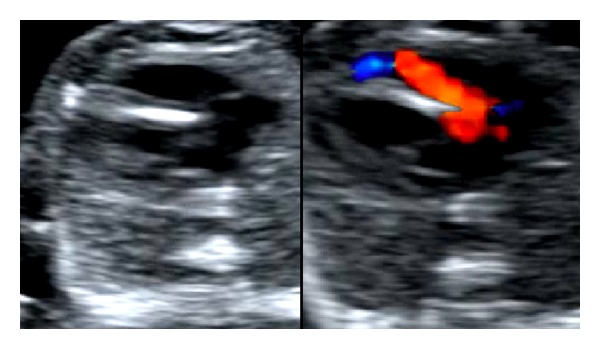
Unilateral perfusion of right ventricle in fetus with hypoplastic left heart syndrome.

**Figure 3:**
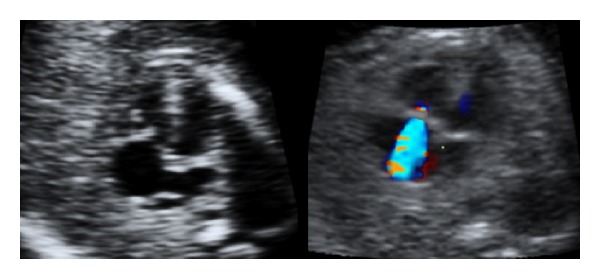
Displacement and insufficiency of the tricuspid valve (Ebstein's anomaly).

In a secondary-level unit the most important objective is not to give a precise diagnosis, but spotting an anomaly. Thus, the results of the ultrasound examinations from our hospital (first column in Table 1) focus on the ultrasound findings and not on an exact diagnosis. Fetuses with suspected fetal heart defects should be referred to a tertiary centre for an extended examination [26]. Whenever a ductus-arteriosus-dependent heart defect is suspected, delivery at a hospital with heart surgery should be planned. In this study we aimed to focus on the main ultrasound finding leading to the detection of an abnormal heart. An unbalanced four-chamber view, a common AV valve, and an abnormal position of the heart are findings mainly related to gray-scale imaging (8/26). Unilateral perfusion of one ventricle, AV insufficiency, and retrograde flow in one of the vessels in the “three-vessel view” are findings related to the use of color Doppler (9/26). Overriding vessel, parallel vessels, and “one great vessel” in the three-vessel view might be detected in gray-scale (9/26); however, these abnormalities are probably also easier to spot using color Doppler.  Figure 1 illustrates a normal-looking gray-scale acquisition of the three-vessel view; however, using color Doppler retrograde flow in the pulmonary artery is obvious.  Figure 2 illustrates unilateral perfusion of the right ventricle in a fetus with hypoplastic left heart syndrome (HLHS). The displacement of the tricuspid valve seen in Figure 3 is detectable in grey-scale; however, the tricuspid insufficiency is even easier to spot.

We missed prenatal diagnosing of a major heart defect in thirteen cases, and five of these children were in a serious condition shortly after birth (two cases with IAA, two with coarctation of the aorta (CoA), and one with TGA). All of them were immediately referred to a center for cardiac surgery and successfully operated. Retrospectively, we think the diagnoses in the seven first cases presented in Table 2 have great importance for the newborn child, and the first five should be possible to detect prenatally. The routine second trimester scan in Norway is recommended between week 17 and 20. A detailed fetal heart scanning is easier later in the second trimester. CoA is difficult to diagnose prenatally, and malformations related to the aortic arch are the most commonly undiagnosed severe heart defects [27]. Unfortunately we missed most of aortic arch malformations in our study, and a method detecting these abnormalities is highly desirable. Small VSDs and moderate pulmonary stenosis are also commonly undiagnosed at delivery; however, these malformations are usually not life threatening shortly after birth. The high survival rate in live born children in our study may be related to high prenatal detection rate and to termination of pregnancy in fetuses with the most critical anomalies. In 10 cases the karyotype was abnormal, but there were only three cases with trisomy 21. This last number was unexpectedly low and might be related to first trimester ultrasound examinations of high-risk women.

A possible disadvantage of using color Doppler is that all extra assessments are time consuming and routine use of color Doppler will have impact on workload in a fetal medicine unit.

We conclude that routine use of color Doppler in fetal heart scanning in a low-risk population may be helpful in the detection of major heart defects; however, still severe malformations are missed prenatally.

## Figures and Tables

**Table 1:** Main ultrasound finding, secondary findings, final diagnosis, and associated anomalies and outcome in 26 fetuses with major heart anomalies.

Main ultrasound finding Ultrasound diagnosis	Final heart diagnosis	Karyotype	Associated anomalies	Time of detection	Outcome
*Unequal size of ventricles*					
Pulmonary stenosis, VSD	Tetralogy of Fallot			Routine	Surgical repair
Retrograde flow in aorta, tricuspid insufficiency	Hypoplastic aortic arch, CoA			Late	Surgical repair
Septum defect, one great vessel	AVSD, pulmonary atresia			Routine	Surgical repair
One great vessel, overriding artery, VSD	Tetralogy of Fallot	Trisomy 13		Late	Died after birth

*Unilateral perfusion of right ventricle*					
One great vessel in “three-vessel view,” HLHS	HLHS			Routine	TOP (no autopsy)
Retrograde blood flow in aorta, HLHS	HLHS			Routine	TOP
One outlet vessel, HLHS	HLHS			Early	TOP

*Common atrioventricular valve*					
Septum defect, atrioventricular insufficiency	AVSD	Trisomy 21		Routine	TOP
Septum defect	AVSD	Trisomy 21	Duodenal atresia	Late	Surgical repair
Septum defect	VSD	Trisomy 18	Multiple defects	Early	TOP

*Atrioventricular insufficience*					
Tricuspid insufficiency, retrograde blood flow in DA, VSD	Ebstein's anomaly			Routine	TOP
Tricuspid insufficiency, retrograde blood flow in DA	Ebstein's anomaly			Routine	TOP (no autopsy)
Tricuspid insufficiency	PA		TTS	Routine	Surgical repair
Mitral insufficiency, septum defect, reduced contractility	ASD, cardiomegaly		Agenesis of kidneys	Routine	TOP

*Overriding vessel*					
VSD	Tetralogy of Fallot			Routine	Surgical repair
VSD	Overriding aorta, ASD, VSD	Trisomy 18	Clinched fingers	Late	IUFD

*Parallel vessels*					
TGA, DORV, VSD, tricuspid insufficiency	DORV, TGA, VSD, PA			Routine	Surgical repair
TGA, DORV, VSD	DORV, TGA			Routine	Surgical repair
TGA, large VSD	TGA, single ventricle		Kyphoscoliosis	Routine	TOP

*One great vessel in “three*-*vessel view” *					
Overriding vessel, VSD	DORV, ASD, VSD	Trisomy 21		Routine	Surgical repair
VSD, tiny pulmonary artery	VSD, pulmonary stenosis			Routine	Surgical repair
Tricuspid insufficiency	DORV, TGA, VSD	Trisomy 18	Clinched fingers	Routine	TOP
No other findings	DORV, TGA			Routine	TOP (no autopsy)

*Retrograde blood flow in “three*-*vessel view” *					
Aortic stenosis, retrograde flow in aorta	Aortic stenosis, VSD			Routine	Surgical repair
Retrograde flow in DA	HRHS, PA			Routine	TOP

*Abnormal position of the heart*					
Heart outside thorax	Ectopic heart, VSD		Kantrell's pentalogy	Early	TOP

VSD: ventricular septal defect; CoA: coarctation of the aorta; ASD: atrial septal defect; AVSD: atrioventricular septal defect; PA: pulmonary atresia; DA: ductus arteriosus; TGA: transposition of the great arteries; DORV: double-outlet right ventricle; HLHS: hypoplastic left heart syndrome; HRHS: hypoplastic right heart syndrome; TTS: twin-twin transfusion syndrome; TOP: termination of pregnancy; IUFD: intrauterine fetal death; routine: detected at a second trimester routine scan; early or late: detected before or after the routine scan.

**Table 2:** Children with major heart defects not detected prenatally.

Diagnosis	Karyotype	Associated anomalies	Outcome
Tetralogy of Fallot			Surgical repair
Transposition of the great arteries			Surgical repair
Double-outlet right ventricle, VSD	Trisomy 13	Agenesis of corpus callosum and cleft lip/palate	Died three days after delivery
Aortic valve stenosis and IAA			Surgical repair
IAA, aorta stenosis, VSD	22q11.2 deletion		Surgical repair
Coarctation of the aorta			Surgical repair
Coarctation of the aorta			Surgical repair
Atrioventricular septal defect	Normal		IUFD
Ventricular septal defect	Trisomy 18	Horseshoe kidney	IUFD
Ventricular septal defect, mild pulmonary stenosis			Surgical repair
Pulmonary stenosis, VSD		Bilateral pes equino varus	Invasive balloon dilatation
Pulmonary stenosis, supravalvular aorta stenosis			Invasive balloon dilatation
Pulmonary stenosis			Invasive balloon dilatation

IAA: interrupted aortic arch; VSD: ventricular septal defect; IUFD: intrauterine fetal death.

## Abbreviations

VSD: Ventricular septal defect
ASD: Atrial septal defect
AVSD: Atrioventricular septal defect
PA: Pulmonary atresia
DA: Ductus arteriosus
TGA: Transposition of the great arteries
IAA: Interrupted aortic arch
CoA: Coarctation of the aorta
DORV: Double outlet right ventricle
HLHS: Hypoplastic left heart syndrome
HRHS: Hypoplastic right heart syndrome
TTS: Twin-twin transfusion
TOP: Termination of pregnancy
IUFD: Intrauterine fetal death
ISUOG: International Society of Ultrasound in Obstetrics and Gynecology
AIUM: American Institute of Ultrasound in Medicine.
